# Electronic-Nose as Non-destructive Tool to Discriminate “Ferrovia” Sweet Cherries Cold Stored in Air or Packed in High CO_2_ Modified Atmospheres

**DOI:** 10.3389/fnut.2021.720092

**Published:** 2021-09-21

**Authors:** Rosaria Cozzolino, Maria Cefola, Carmine Laurino, Mario Paolo Pellicano, Michela Palumbo, Matteo Stocchero, Bernardo Pace

**Affiliations:** ^1^Institute of Food Science, National Research Council (CNR), Avellino, Italy; ^2^Institute of Sciences of Food Production, CNR-National Research Council of Italy, Foggia, Italy; ^3^Department of Science of Agriculture, Food and Environment, University of Foggia, Foggia, Italy; ^4^Department of Women's and Children's Health, University of Padova, Padova, Italy

**Keywords:** sweet cherry cv “Ferrovia”, electronic-nose (E-nose), volatile profiles, projection to latent structure, correlation analysis, firmness

## Abstract

This study aimed to explore the applicability of electronic-nose (E-nose) as a rapid method in discriminating samples of sweet cherry cv “Ferrovia” stored in high-CO_2_ (16% O_2_ + 20% CO_2_ + 64% N_2_) or air (control) up to 21 days. Projection to Latent Structures (PLS) methods applied to E-nose data showed that fresh fruit and the packaged or unpackaged samples can be distinguished, according to both the storage condition and the storage days. Moreover, a correlation analysis between E-nose sensors and 45 volatile compounds were overall, obtained from all the investigated sweet cherry samples by Headspace Solid-Phase Microextraction (HS SPME) coupled to Gas Chromatography-Mass Spectrometry (GC-MS). These methods allowed to associate samples with a specific flavour profile to one or more E-nose sensors. Finally, quality attributes (visual quality, colour, firmness, antioxidant activity, total phenols, and sugar content) were assessed during storage. Among these, visual quality and berry deformation resulted affected by storage conditions, showing that high-CO_2_ treatment better preserved the fruit quality than control.

## Introduction

Cold temperatures storage and modified atmosphere (MA) packaging are well-established practises for delaying the senescence of fresh fruit and vegetables, extending their postharvest life.

Aroma and flavour, which are crucial sensory attributes for consumer acceptability, are directly influenced by the organic volatile compounds (VOCs) profiles (1). Volatile metabolites represent the final products of fruit metabolism and changes in their profiles during storage can suggest the development of spoilage (2). In the last decades, the identification and quantification of the aroma compounds by Headspace Solid-Phase Microextraction (HS SPME) sampling followed by Gas Chromatography-Mass Spectrometry (GC–MS) became a well-established method for the evaluation of the most suitable postharvest conditions to preserve the sensory quality of freshly harvested fruit and vegetables during the storage (3). However, the practical application in the food industry of HS SPME and GC-MS is limited because these techniques involve expensive instrumentations and need considerable analytical skilled manpower to handle the complex mixture and the high number of volatiles that comprise the VOCs pattern of crops (4). In recent years, electronic nose (E-nose) technology has been demonstrated to overcome some of the drawbacks associated with the methods traditionally used for the evaluation of VOCs profile (3). Electronic nose is a device equipped with an array of partial specific and broad-spectrum electronic chemical sensors which simulate human olfactory perception and offers a digital fingerprint of the volatile components that can be investigated with appropriate statistical tools. The use of the olfactometric method for the investigation of volatiles of fruit and vegetable presents several advantages, such as low cost, easy-to-handle, fast and non-destructive analysis, no preliminary sample preparation steps, and automatic data management (5). However, there are some disadvantages of using different E-nose sensors, including their response and recovery times, sensitivities, detection range, operating restrictions, physical size, inactivation by certain poisoning agents, and other limitations that are specific to individual sensor types. As clearly reported by the study of Wilson and Baietto (6), the varieties of advantages and limitations related to each E-nose sensor type are strictly connected with the nature of the technology that regulates the principle for detection and the types of analytes that may be detected by each sensor type. This technique has already an application in the food industry, including food quality control, authenticity, and traceability studies of fresh fruit (4).

Sweet cherries are greatly appreciated worldwide because of their appealing colour, aroma, taste, and health beneficial nutritional properties. This fruit is very perishable, and for the various cultivars, different MA treatments have been reported to preserve sweet cherry quality for the fresh market (7–10). A typical Italian sweet cherry cultivar “Ferrovia” is very appreciated for its big and bright skin berries, characterised by agreeable and middle sweetness flavour that allows this cultivar to be suitable for fresh consumption (11). In a very recent study of Cozzolino et al. (12), the changes of VOCs profiles were detected by HS SPME and GC-MS of sweet cherries cv “Ferrovia,” cold-stored in high CO_2_ modified atmospheres with three different compositions up to 21 days (11). Among the MA treatments, the authors observed that storage in high-CO_2_ (16% O_2_ + 20% CO_2_ + 64% N_2_) resulted in the most suitable packaging condition in preserving the sensory quality of the fruit till the end of storage (12).

The objective of the present study was to explore, for the first time, the applicability of E-nose as a rapid methodology in differentiating samples of sweet cherry cv “Ferrovia” stored in high-CO_2_ or air (control) for up to 21 days. Multivariate and univariate statistical data analysis have been applied to investigate the effects of storage time and storage condition on the E-nose fingerprint and VOCs profile [previously studied in (12)], while correlation analysis was performed to discover the relationships between sensors and VOCs. Moreover, some quality attributes, including visual quality (VQ), colour, deformation, antioxidant activity, total phenols, and sugar content, have also been investigated.

## Materials and Methods

### Chemicals and Reagents

Sodium carbonate was purchased from Merck (Germany), while 2,2-Diphenyl-1-picrydazyl (DPPH), hydrochloric acid, potassium chloride, sodium acetate, and Folin-Ciocalteu's phenol reagent were obtained from Fluka (Buchs, Switzerland).

### Sweet Cherry Samples

Sweet cherries (*Prunus avium* L., cv Ferrovia) were provided by a local farm (Ermes snc, Noicattaro, Italy) in May 2017 and immediately transported to the Postharvest Laboratory of ISPA CNR. Fruits weighing 200 g were closed in 30 cm × 40 cm polyamide/polyethylene (PA/PE, 90μm thick, Orved, Musile di Piave, Italy) plastic bags (Boxer 50, Lavezzini Vacuum Packaging System, Fiorenzuola d'Arda, Italy) in a modified atmosphere of high-CO_2_ (16% O_2_ + 20% CO_2_) or were stored in open bags in air (control). All samples in triplicate were stored at 5°C and were analysed, as reported below, at harvest (fresh sample) and after 14 and 21 days. The headspace gas composition (O_2_ and CO_2_) within each high-CO_2_ bag was measured every day using a gas analyser (CheckPoint, PBI Dansensor, Ringsted, Denmark) and a gas chromatograph (p200 micro-GC, Agilent, CA, USA).

### Quality Analysis

In order to evaluate the effect of storage on the visual quality, the fruits were scored by 10 trained panellists, using a hedonic scale of 5–1, with 5 = excellent, no defects; 4 = very good, minor defects; 3 = fair, moderate defects (limit of marketability); 2 = poor, major defects (limit of edibility) and 1 = inedible (13).

The L^*^ (lightness), a^*^ (redness), and b^*^ (yellowness) parameters were measured at 3 random points on 10 fruits for each replication, using a colorimeter (CR-400, Konica Minolta, Osaka, Japan), as previously described (14). The ΔE^*^ was then calculated from primary a^*^ and b^*^ readings, using the following formula $\Delta {\text{E}}^{*}=\left[{\left(L_{0}^{*}-L^{*}\right)}^{2}+{\left(a_{0}^{*}-a^{*}\right)}^{2}+{\left(b_{0}^{*}-b^{*}\right)}^{2}\right]1/2$ (15).

Sweet cherry deformation was measured, on 10 fruits from each replication, as the force required to obtain a 3 mm deformation of each berry, using a texture analyser (ZwickLine Z0.5-Zwick/Roell, Ulm, Germany) equipped with a 100 mm diameter plate (16). The measure was expressed in %, normalising the diameter of the berry.

The total soluble phenolic compounds were analysed on fresh weight according to the Folin-Ciocalteu colourimetric method (17) and the concentrations were expressed in mg g^−1^ of gallic acid equivalents. The antioxidant activity was assessed on fresh weight by using the DPPH assay (18).

Total sugars were analysed on ethanol extracts by a phenol-sulfuric colourimetric method (19) with colour development at 490 nm and glucose as a standard.

### Analysis of Volatile Compounds

Volatile compounds identification and semi-quantification by HS SPME and GC-MS were previously reported in the study of Cozzolino et al. (12) which used DVB/CAR/PDMS (50/30 μm) fibre at 45°C and 20 min as extraction temperature and time, respectively. Volatiles were analysed by using the GC device, model GC 7890A, Agilent (Agilent Technologies, CA, USA), coupled to the mass spectrometer 5975 C (Agilent). Semi-quantitative data of each metabolite (Relative Peak Area, RPA%) were calculated with respect to the peak area of 4-methyl-2-pentanol, used as IS (12). Areas of the identified metabolites were measured from the total ion chromatogram (TIC).

### Evaluation of Cherry Samples by E-Nose

The aroma profiles from the headspace of cherry samples were carried out using a commercial portable E-nose (E-nose, PEN 3, Airsense Analytics Inc., Schwerin, Germany, including the Win Muster software). The electronic nose system consists of a sampling unit and a gas detection system equipped with an array of 10 metal oxide semiconductors (MOS) sensors with different thicknesses and chemical compositions, to offer selectivity toward various volatile classes, as reported in the study of Shi et al. (20). Due to the high operative temperatures (200–500°C), VOCs transported to the surface of the sensors were completely combusted to carbon dioxide and water, causing a change in the resistance. The response of the MOS sensors, expressed as resistivity (O), was based on the variations in conductivity, due to the adsorption of gas molecules, and on the following surface reactions. For sample preparation, 4 g of each sample were put in 45 ml airtight glass vials and sealed with a screw cap with Poly (1,1,2,2-tetrafluoroethylene) (PTFE)/silicone septum. To reach the headspace equilibrium, each vial was kept at 30°C for 30 min and analysed at 22 ± 2°C and 50 ± 5% relative humidity (RH). In the course of the measurement time, the gas headspace was injected into the E-nose for 80 s at 400 ml/min. Analyses were conducted in six technical replicates for each biological sample and data were collected at each second. When the volatiles reached the measurement unit, the sensor conductivity modifies, first growing and then stabilising reaching a steady-state, causing changes of the ratio G/G0 (G and G0 are conductance of the sensors exposed to sample gas and zero gas, respectively) of each sensor. Subsequently, a second pump carries the filtered air to the sensor array for 400 s with a flow rate of 600 ml/min to clean the system between two consecutive analyses.

Electronic nose data were collected by the pattern recognition software (WinMuster, v.1.6., Airsense Analytics GmbH, Germany) and the average of each sensor response in the range from 70 to 75 s (area under the curve) was used for statistical data analysis.

### Statistical Data Analysis

Quality parameters were analysed by ANOVA (Statistica 6 software, SatSoft Inc, Tulsa Oklahoma), considering factors such as storage condition (high-CO_2_ or air), storage days (14 and 21 days), and their interaction.

The electronic nose data and the VOCs have been investigated by ANOVA and multivariate data analysis based on projection methods. Specifically, the factors storage condition, storage days, and their interaction term have been included in the ANOVA model controlling the false discovery rate by the Benjamini-Hochberg procedure at the level δ = 0.05. Principal Component Analysis (PCA) has been applied for exploratory data analysis whereas Projection to Latent Structure (PLS) by partial least squares regression were conducted to investigate the effects of storage condition and storage days on the collected data (21).

The relationships between E-nose responses and VOCs have been explored by correlation analysis. Specifically, the Pearson correlation coefficient has been calculated for all pairs of E-nose data and VOCs and the resulting correlation matrix has been investigated by heatmap using a clustering procedure based on Euclidean distance and Ward's method.

Data analysis has been performed by in house R-function implemented using the R 4.0.4 platform (R Foundation for Statistical Computing, Vienna, Austria).

## Results and Discussion

### Changes in Quality Parameters of Sweet Cherries Cold Stored in High-CO_2_ or Air

**Figure 1:**
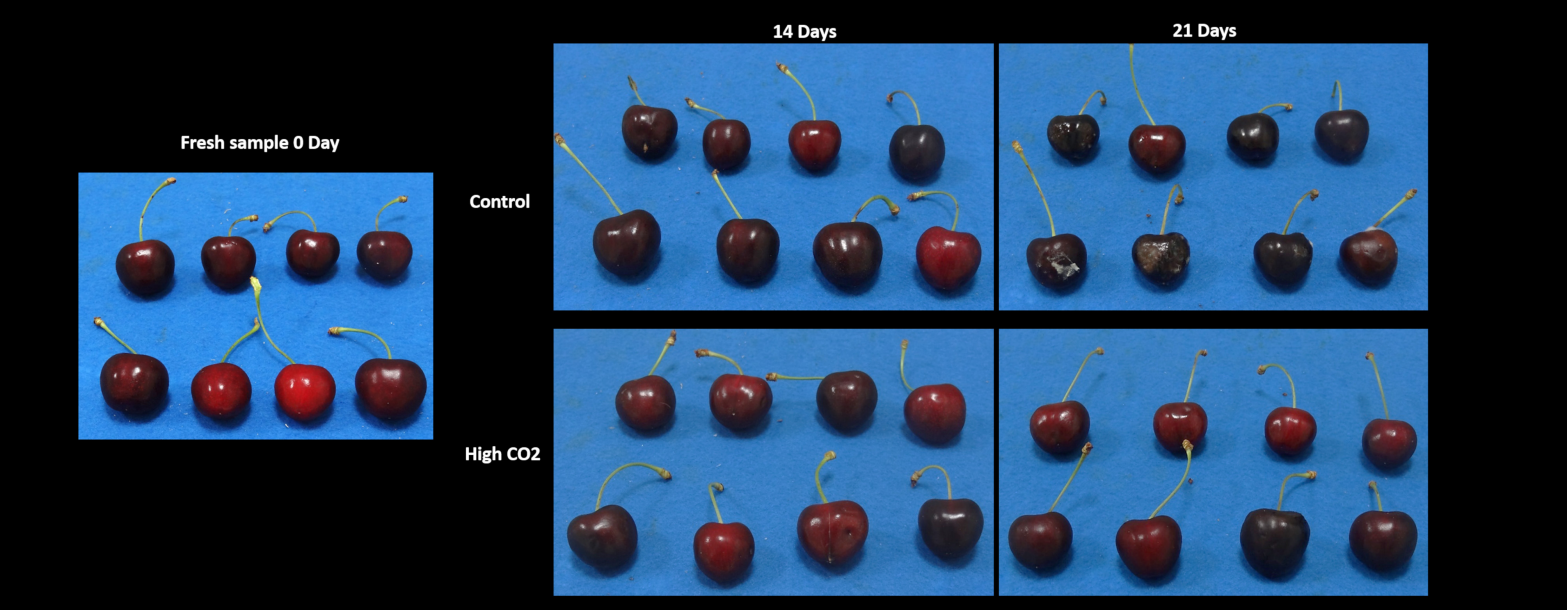
The visual appearance of sweet cherries at harvest and stored in high-CO_2_ or the air (control) after 14 and 21 days at 5°C.

Fruit stored in high-CO_2_ reported a mean value of the visual quality significantly higher than control, with a quality decrease during storage from 5 to roughly 2.5 at the end of the storage (Figure 1, Table 1). Changes in visual quality during storage can be mainly related to the browning of the skin and a significant increase in berry deformation (Table 1). This last parameter resulted higher in air than in high-CO_2_ fruit, influencing the sensory evaluation of the visual quality. This result confirmed the positive effect of high-CO_2_ MA storage in the preservation of quality of sweet cherry cv. “Ferrovia,” as previously reported in the study by Cozzolino et al. (12).

**Table 1:** Main effects of storage days and storage condition on visual quality score, ΔE^*^, fruit deformation and antioxidant activity of sweet cherries (cv. *Ferrovia*).

	**Visual quality (score 5-1)**		**ΔE** ^*^		**Fruit deformation (%)**		**Antioxidant activity (mg Trolox/100 g fw)**	
**Days**	^**^		^*^		^**^		^*^	
14	3.0	b	5.7	ab	12.4	b	69.74	b
21	2.5	c	7.4	a	14.1	a	85.29	a
**Storage**								
**condition**	^***^		ns		^***^		ns	
Control	3.0	b	6.3		13.9	a	76.35	
High-CO_2_	4.5	a	5.0		12.3	b	78.56	

*For each parameter, different letters indicate a statistical difference for p = 0.05 (LSD test). Ns, not significant*;

* *p ≤ 0.05*;

** *p ≤ 0.01*;

*** *p ≤0.001. Data are mean values of 3 replicates for each storage condition at each storage day*.

For fresh fruit the initial mean values of antioxidant activity, total phenols and total sugar content were 63.6 ± 7.2 mg Trolox 100 g^−1^ fw, 130.3 ± 6.2 mg gallic acid 100 g^−1^, and 21.04 ± 2.0 g glucose 100 g^−1^, respectively. Except for the antioxidant activity that significantly increased during the storage of about 25% in both storage condition, the other parameters were kept unchanged during the experiment and were not affected by the storage condition (Table 1).

### Changes in E-Nose Responses of Sweet Cherries Cold Stored in High-CO_2_ or Air

Five biological samples have been collected for fresh produce and each combination of storage condition (air or high-CO_2_) and storage days (14 and 21 days at 5°C). Since six technical replicates have been considered for each biological sample, a data set composed of 150 observations was obtained.

**Figure 2:**
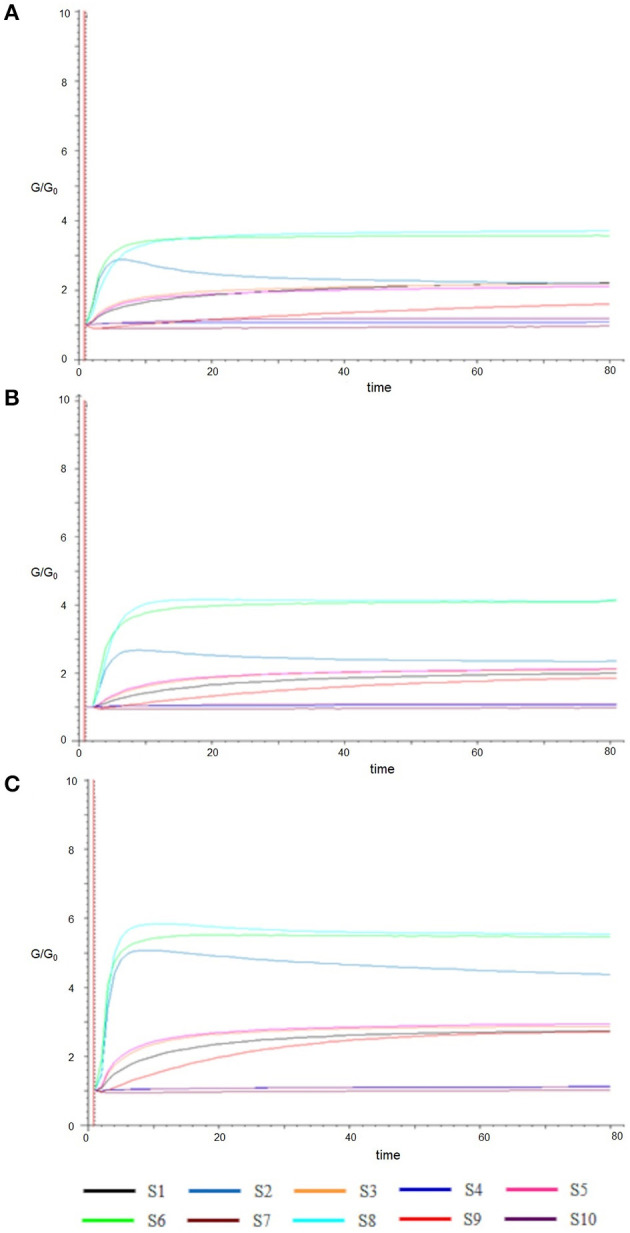
Typical responses of sensors (S1–S10) to Fresh **(A)** and samples cold stored in Air **(B)** and High CO_2_ **(C)** at 21 days.

In Figure 2, the typical E-nose responses to Fresh (A) and cold-stored sweet cherry samples in air (B) or high-CO_2_ (C) at 21 days are represented. Each curve displayed the response values, expressed as the ratio G/G0 (vertical axis) of the relative sensor which changed with time (horizontal axis). The signals trend in all samples was almost similar. The response values of S8 (sensitive to broad-alcohol) appeared significantly increased, followed by S6 (sensitive broad-methane) and S2 (broad range). On the contrary, the signals of the sensors S4, S6, and S10 were sensitive to hydrogen, broad-methane, and methane-aliph, respectively. They did not show significant variations, keeping the value of G/G0 around or less than one. Finally, the sensors S1, S3, S5, and S9, which were all responsive to aromatic components, regularly declined slightly. Comparing the three panels of Figure 2, the values of S8, S6, and S2 were different and indicate that sweet cherries stored in high-CO_2_ (C) could produce more aroma components than fresh (A) and air (B) samples.

**Figure 3:**
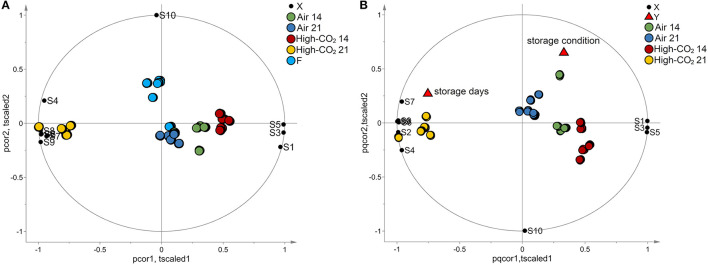
E-nose data. In **(A)**, the biplot generated by PCA is reported. The points representing the E-nose sensors (black circles) are reported in the same plot of the points representing the samples of sweet cherry cv “Ferrovia” investigated in our study. Fresh samples (light blue circles) spread along the vertical axis mainly influenced by sensor S10 whereas the other samples spread along the horizontal axis. Specifically, samples with the same storage days and storage conditions are represented by points close together in the plot. In **(B)**, the biplot obtained by PLS analysis is reported. The points representing the E-nose sensors (black circles), the points representing the responses “storage days” and “storage condition” (red triangles), and the points representing the samples are reported in the same plot. Samples with the same storage days and storage conditions are represented by points close together in the plot. Moreover, responses and sensors positively correlated are represented by points close to each other while if they are inversely correlated, the points are centrosymmetric. The effect of storage condition is mainly explained by the behaviour of the S10 sensor whereas the data variation of the other sensors is mainly related to the effect of the storage days.

The data set composed of 150 observations and 10 features has been autoscaled and investigated by PCA. A model with two principal components explaining 97% of the total variance has been obtained. The biplot of the model is reported in Figure 3A. Fresh fruits were spread along the second component being characterised by high responses of the sensor S10, whereas the other observations were spread along with the first component. The responses of the sensors S1, S3, and S5 had decreased from 14 to 21 days, while the responses of sensors S2, S4, S6, S7, S8, and S9 showed an opposite trend. Observations with the same storage days and storage conditions were represented by points close together in the plot.

To better investigate the effect of the storage condition and the storage days on the E-nose responses, a PLS model has been built considering the storage condition and the time of storage as responses and the E-nose data as predictors. In the analysis, the fresh fruits were excluded. Considering autoscaled data, a PLS model with 3 latent variables, R^2^Y = 0.75 and Q^2^Y = 0.59 has been obtained. The model passed the permutation test on the responses (1,000 random permutations). The biplot of the model is reported in Figure 3B. The effect of storage condition was mainly explained by the behaviour of the S10 sensor whereas the data variation of the other sensors was mainly related to the effect of the storage days. The trend discovered by PCA for the responses of sensors S1, S2, S3, S4, S5, S6, S6, S7, S8, and S9 was confirmed by PLS analysis.

Projection to latent structure findings were also confirmed by ANOVA performed on the E-nose data, which highlighted that all the sensors are significantly affected by storage condition and storage days except for S10 that is influenced only by storage condition (Table 2).

**Table 2:** ANOVA: *p*-value (*p*) of the effects of storage condition (High-CO_2_ and Air), storage days (14 and 21 days at 5°C), and their interaction on VOCs and E-nose data. Effects selected by Benjamini-Hochberg procedure assuming δ = 0.05 are indicated with “*”.

**Type**	**Name**	**Code**	**p[storage condition]**	**p[storage days]**	**p[interaction]**
**VOCs**					
**Esters**					
	Ethyl acetate	E1	3.5E-05^*^	3.5E-05^*^	3.5E-05^*^
	Ethyl 2-butenoate	E2	1.1E-01	1.1E-01	1.1E-01
	Ethyl Hexanoate	E3	1.2E-02	1.2E-02	1.2E-02
	1-Hexyl acetate	E4	5.3E-01	6.8E-01	6.3E-01
	2-Hexen-1-ol acetate	E5	8.5E-01	2.6E-01	6.8E-01
	2-Hexenyl butyrate	E6	6.8E-01	1.0E-03^*^	3.4E-02
	Ethyl benzoate	E7	2.4E-02	2.4E-02	2.4E-02
	trans 2-hexenyl hexenoate	E8	2.4E-01	2.4E-01	2.4E-01
	2-Hexenyl tiglate	E9	1.7E-02	5.1E-01	5.1E-01
	Isopropyl Laurate	E10	2.5E-03^*^	4.8E-01	4.8E-01
**Alcohols**					
	1-Penten-3-ol	Al1	2.8E-02	2.7E-02	7.4E-02
	3 Hexanol	Al2	1.6E-02	1.6E-02	1.6E-02
	1 Pentanol	Al3	1.2E-04^*^	3.3E-03^*^	3.3E-03^*^
	3-Methyl-3-buten-1-ol	Al4	8.0E-01	4.3E-01	8.2E-01
	3-methyl-2-Buten-1-ol	Al6	1.8E-01	4.4E-01	9.6E-01
	1-Hexanol	Al7	3.0E-01	8.4E-01	8.8E-01
	trans 3-Hexen-1-ol	Al8	1.7E-01	3.1E-01	3.9E-01
	cis 3-Hexen-1-ol	Al9	1.0E+00	1.2E-01	8.7E-01
	cis 2-Hexen-1ol	Al10	8.5E-01	4.7E-01	9.8E-01
	1-Octanol	Al11	6.6E-01	3.0E-01	7.0E-01
	Nonanol	Al12	8.1E-01	1.3E-01	1.8E-01
	Benzene methanol	Al13	7.4E-01	3.2E-01	6.4E-01
	1-Dodecanol	Al14	7.8E-01	5.4E-02	7.9E-01
**Aldehydes**					
	Butanal 3-methyl	Ald1	1.7E-01	1.7E-01	1.7E-01
	Hexanal	Ald2	4.1E-01	3.6E-01	8.3E-01
	2-Hexenal	Ald3	4.0E-01	4.4E-01	8.0E-01
	Octanal	Ald4	1.1E-01	3.9E-02	3.9E-01
	Nonanal	Ald5	7.4E-01	7.4E-02	8.0E-01
	Decanal	Ald6	3.0E-02	7.1E-01	7.1E-01
	Benzaldehyde	Ald7	1.4E-01	8.3E-01	8.6E-01
	Dodecanal	Ald8	4.8E-01	5.6E-01	8.8E-01
	Tetradecanal	Ald9	4.5E-02	1.8E-01	1.2E-01
**Ketones**					
	2 pentanone 4 methyl	K2	4.9E-02	1.6E-01	5.8E-01
	γ Butyrolactone	K4	6.4E-07^*^	6.4E-07^*^	6.4E-07^*^
	2-Dodecanone	K5	7.4E-03^*^	5.6E-01	5.6E-01
**Terpenes**					
	dl-Limonene	T1	3.5E-03^*^	8.9E-01	8.9E-01
	Linalool	T3	8.3E-01	2.9E-01	3.1E-01
	α Terpineol	T4	6.0E-01	4.7E-02	2.1E-02
**Others**					
	Formammide N,N-dibutyl	O2	7.2E-01	5.5E-01	7.4E-01
	Benzothiazole	O3	3.3E-01	5.5E-01	8.9E-02
**E-nose sensors**					
	S1		2.3E-07^*^	2.3E-12^*^	4.7E-11^*^
	S2		8.5E-10^*^	9.8E-13^*^	1.2E-11^*^
	S3		1.9E-06^*^	9.2E-13^*^	3.5E-11^*^
	S4		4.4E-09^*^	3.2E-10^*^	5.2E-09^*^
	S5		1.4E-05^*^	8.2E-13^*^	1.1E-10^*^
	S6		2.4E-07^*^	1.7E-13^*^	5.2E-09^*^
	S7		7.3E-03^*^	1.3E-09^*^	2.4E-09^*^
	S8		8.5E-07^*^	2.9E-13^*^	2.1E-09^*^
	S9		1.9E-08^*^	1.4E-13^*^	3.3E-11^*^
	S10		5.3E-03^*^	1.6E-01	8.2E-01

### Changes in VOCs of Sweet Cherries Cold Stored in High-CO_2_ or Air

Three biological samples have been collected for fresh fruit and each combination of storage condition (air or high-CO_2_) and storage days (14 and 21 days at 5°C) obtaining a data set composed of 12 observations and 45 VOCs.

**Figure 4:**
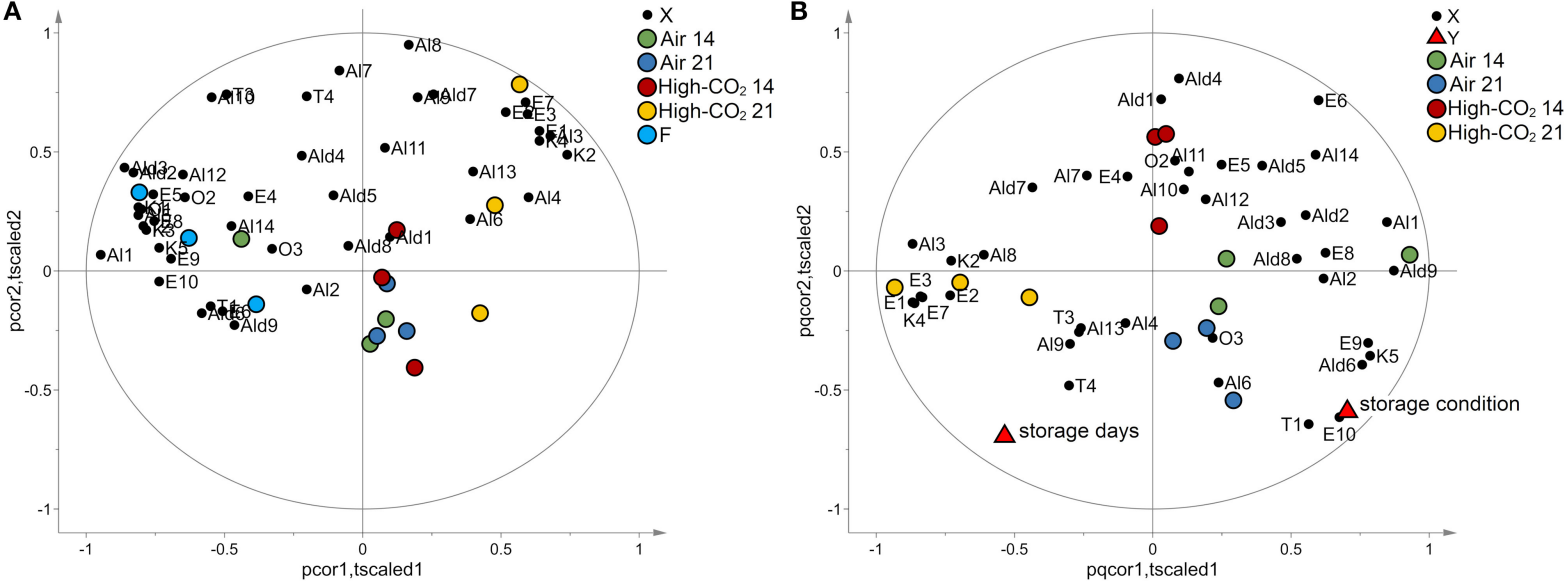
VOCs data. In **(A)**, the biplot generated by PCA is reported. The points representing the VOCs (black circles) are reported in the same plot of the points representing the samples of sweet cherry cv “Ferrovia.” Fresh fruits (light blue circles) and samples packed in high-CO_2_ for 21 days (yellow circles) are evidently separated from the other samples. When VOCs and points representing samples are close, VOCs show high levels for those samples or if their images obtained by inversion in the origin are close to the points representing the samples, low levels. In **(B)**, the biplot obtained by PLS analysis is reported. The points representing the VOCs (black circles), the points representing the responses “storage days” and “storage condition” (red triangles), and the points representing the samples are reported in the same plot. Samples with the same storage days and storage conditions are represented by points close together in the plot. Moreover, responses and VOCs positively correlated are represented by points close to each other while if they are inversely correlated, the points are centrosymmetric.

Applying autoscaling to the data, a PCA model with two principal components has been obtained. The model explained 54% of the total variance. The biplot is reported in Figure 4A. Fresh fruits and samples packed in high-CO_2_ for 21 days were evidently separated from the other samples being characterised by specific VOCs. Sweet cherries stored in air or Hhgh-CO_2_ for 14 days were located in the centre of the plot and are not characterised by specific VOCs.

The effects of storage condition and storage days on the VOCs have been investigated by PLS analogously to the case of E-nose data. Considering autoscaled data, a PLS model with two latent variables, R^2^Y = 0.81 and Q^2^Y = 0.62, has been obtained. The effects of storage condition resulted better explained than in the case of E-nose (R^2^Y = 0.84 and Q^2^Y = 0.67 for VOCs and R^2^Y = 0.55 and Q^2^Y = 0.43 for E-nose data). The model passed the permutation test on the responses (1,000 random permutations). The biplot of the model is reported in Figure 4B. The storage condition affects mainly E1, E3, E7, E9, E10, T1, K4, K5, Ald6, and Al3, whereas E6 is influenced mainly by the storage days.

**Figure 5:**
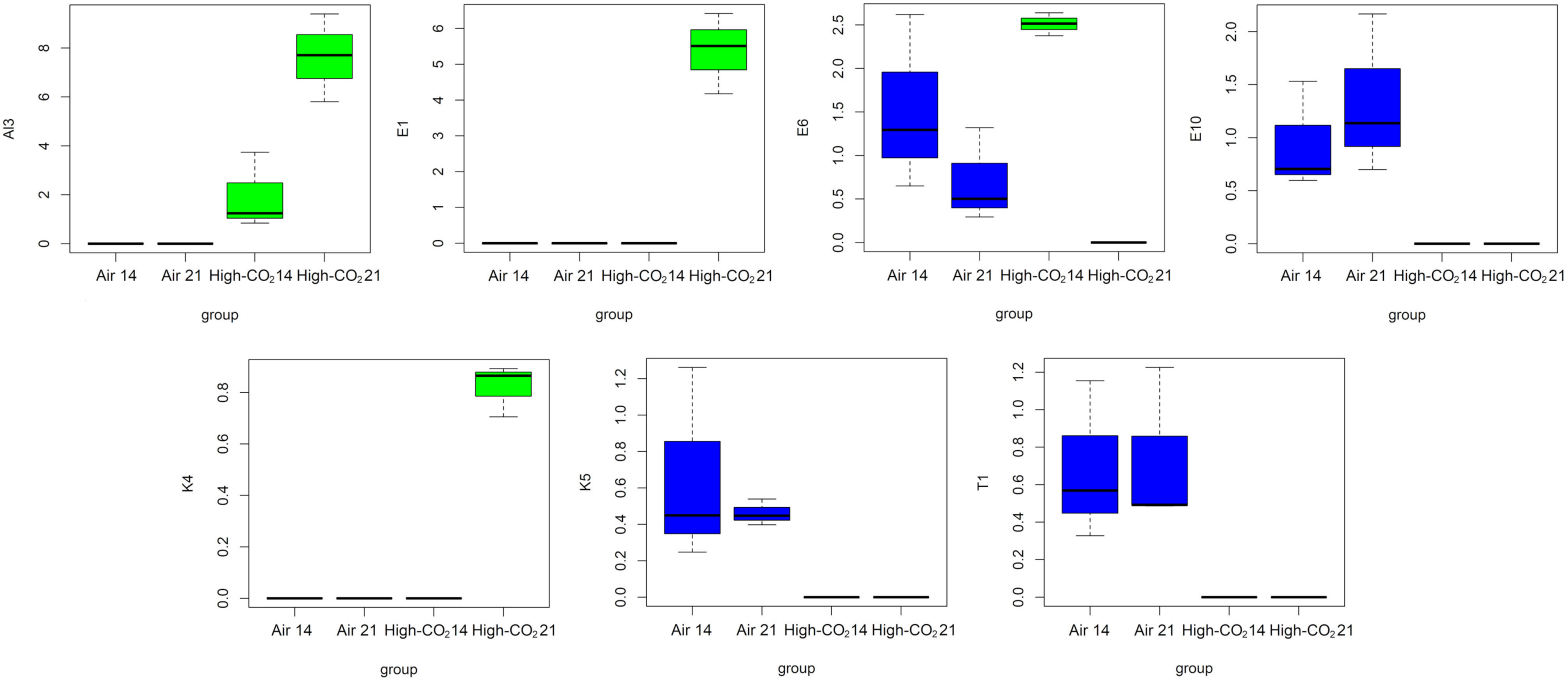
Boxplots representing the distributions of the VOCs highlighted by ANOVA controlling the false discovery rate at the level δ = 0.05.

Analysis of variance performed on VOCs highlighted that E1, Al3, and K4 were significantly affected both by storage condition and by storage days. Moreover, E10, K5, and T1 were significantly affected by storage conditions, and that E6 is significantly affected by storage days (Table 2). The distributions of the selected VOCs are reported in Figure 5.

### Correlation Analysis of E-Nose Data and VOCs

**Figure 6:**
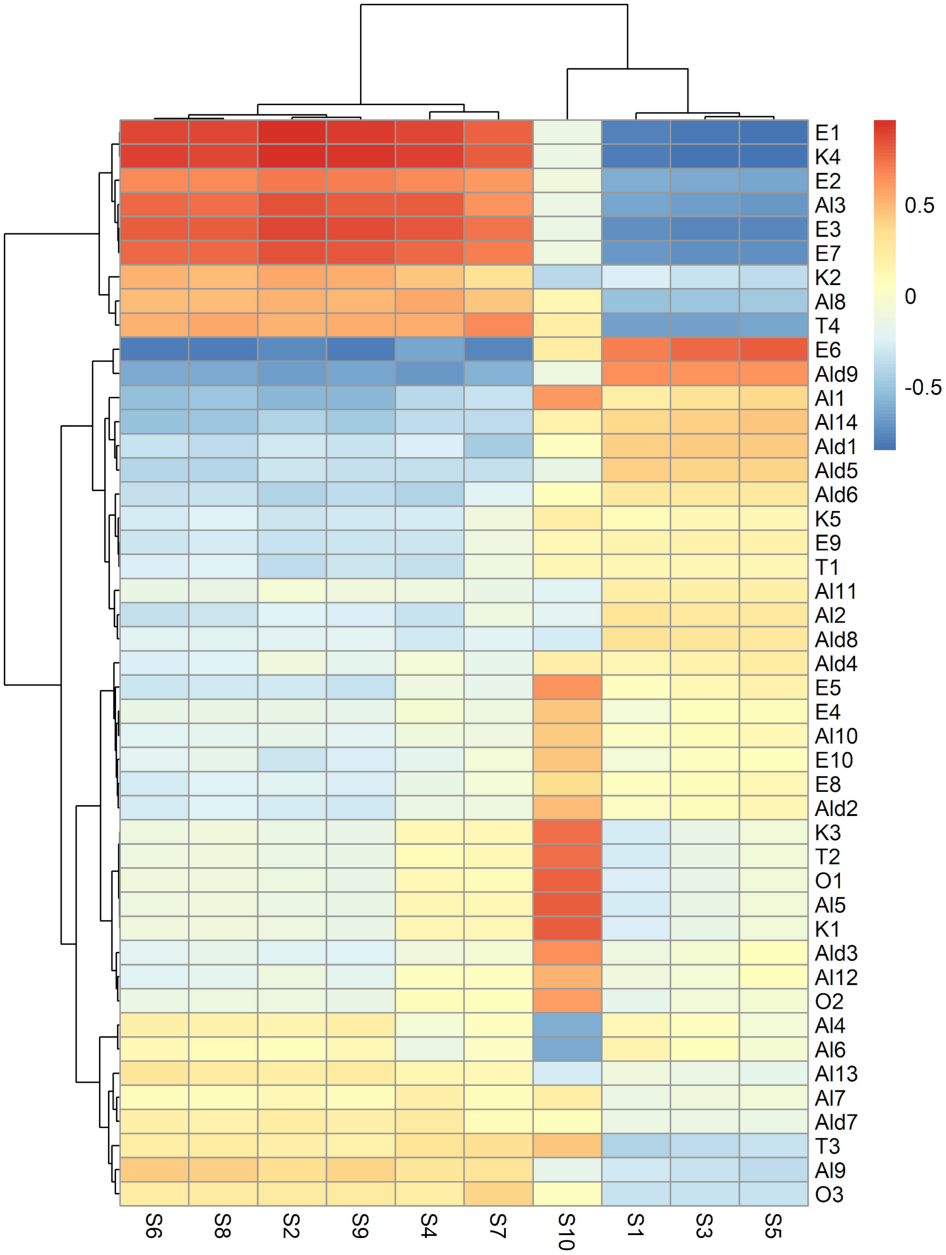
Heatmap representing the correlation between VOCs and E-nose data sensors. In the heatmap, the colour of each cell depends on the value of the Pearson correlation coefficient between the VOC and E-nose data sensor. VOCs and E-nose data have been clustered based on the Euclidean distance and Ward's method. As a result, specific groups of VOCs positively or negatively correlated to specific groups of E-nose sensors can be visually discovered, searching the regions of the map with the highest intensity (red or blue, respectively).

Forty-five VOCs, which were overall identified in the study by Cozzolino et al. (12), were determined in the headspace of fresh, air, and high-CO_2_ samples during the storage (Table S1). These VOCs were correlated with the E-nose sensors data. The heatmap, presented in Figure 6, showed that the data of the sensors S2, S4, S6, S8, S7, and S9 had positive correlations with ethyl acetate (E1), ethyl 2-butenoate (E2), ethyl hexenoate (E3), ethyl benzoate (E7), 1-pentanol (Al3), *trans* 3-Hexen-1-ol (Al8), Terpineol (T4), and γ-Butyrolactone (K4), respectively. The response of the sensors S2, S4, S6, S8, S7, and S9 were in agreement with the VOCs pattern analysis which indicated that high-CO_2_ treatment seems to preserve the quality of the packaged fruit, since ethyl esters and γ-butyrolactone produced by the fermentative metabolism were observed in high-CO_2_ fruit only at the end of the storage (21 days), as reported in Table S1.

The sensor S1 was negatively correlated to *trans* 3-Hexen-1-ol (Al8), while the signals of S2 and S4 were indirectly associated to tetradecanal (Ald9) (Figure 6). These findings demonstrated that S1, S2, and S4 were found to correlate aliphatic compounds, such as esters (E1, E2, E3, and E7), alcohols (Al8), and aldehydes (Ald9).

The signal of the sensor S10 was directly correlated to 14 volatiles, including 1-hexyl acetate (E4), 2-hexen-1-ol acetate (E5), *trans* 2-Hexenyl hexenoate (E8), Isopropyl laurate (E10) *cis* 2-penten-1-ol (Al5), *cis* 2-hexen-1-ol (Al10), Nonanol (Al12), hexanal (Ald2), 2-Hexenal (Ald3) 3-pentanone (K1), 1-penten-3-one (K3), ocymene (T2), 2-methylfuran (O1), and formamide N,N-dibutyl (O2) (Figure 6). These results could suggest that the S10 signal could discriminate potential odorant markers of freshness in “Ferrovia” sweet cherries since all these volatiles were present in the fresh sample. In addition, most of them (Al5, Al10, Ald12, K1, K3, Ald2, Ald3, T2, and O1) have been previously found to be directly correlated to the sensory attribute of *Herbaceous Smell* (11).

On the contrary, the S10 signal exhibited a negative correlation with 3-methyl-3-buten-1-ol (Al4) and 3-methyl-2-buten-1-ol (Al6) (Figure 2). This result supported the above finding, as both the alcohols which are always absent in the fresh fruit, are by-products of alcoholic fermentation and have been previously reported to be inversely related to the sensory trait of *Herbaceous Smell* (12).

The ester 2-hexenyl butyrate (E6) was directly associated with S5 but negatively correlated to S2, S4, S6, S7, S8, and S9. This is in agreement with the fact that E6, in contrast to the volatiles positively correlated to S2, S4, S6, S7, S8, and S9, was detected in the fresh sample, but was completely absent at the end of the storage in high-CO_2_ (Table S1, Figure 2).

The results of the correlation between HS SPME and GC-MS and E-nose data showed that E-nose signal responses were in agreement with the VOCs profile analysis. Additionally, our findings demonstrated that the E-nose could discriminate fresh, air, and high-CO_2_ sweet cherry samples by responding to specific flavour profiles of the samples.

## Conclusion

The present study showed that E-nose is a promising tool in distinguishing sweet cherries cv. “Ferrovia” stored in high-CO_2_ or air (control) for up to 21 days. Indeed, the variations of the response of the E-nose sensors depended both on the condition and the day of storage. The relationships between volatile profiles and E-nose signals have been investigated and the E-nose response has been associated with well-defined sets of VOCs. In particular, the signal of the sensor S10, resulting in association with VOCs which are considered putative markers of freshness, might be used for a rapid assessment of the product quality. Finally, since E-nose data were in agreement with the results previously obtained by HS SPME and GC-MS, commercial E-nose may be used in monitoring changes in aroma fingerprint during fruit storage instead of the expensive laboratory equipment currently used. These features and the non-destructive character of the analysis make E-nose useful for real-time physiological evaluations and quality control for industries in which rapid analysis is required.

## Data Availability Statement

The raw data supporting the conclusions of this article will be made available by the authors, without undue reservation.

## Author Contributions

RC and MC designed experiments. BP, CL, and MP carried out the experiments. RC, MC, and MP analysed the experimental results and wrote the manuscript. MC and MS performed the statistical analysis. All authors contributed to the article and approved the submitted version.

## Funding

The project Prin 2017 MultI Functional polymer cOmposites based on groWn matERials (MI-FLOWER) (Grant Number: 2017B7MMJ5_001) from the Italian Ministry of Education University and Research Project High-Performing Advanced Material Platform for Active and Intelligent Food Packaging is kindly acknowledged.

## Conflict of Interest

The authors declare that the research was conducted in the absence of any commercial or financial relationships that could be construed as a potential conflict of interest.

## Publisher's Note

All claims expressed in this article are solely those of the authors and do not necessarily represent those of their affiliated organizations, or those of the publisher, the editors and the reviewers. Any product that may be evaluated in this article, or claim that may be made by its manufacturer, is not guaranteed or endorsed by the publisher.

## References

[1] Cozzolino R Martignetti A Pellicano MP Stocchero M Cefola M Pace B. Characterisation of volatile profile and sensory analysis of fresh-cut “Radicchio di Chioggia” stored in air or modified atmosphere. Food Chem. (2016) 192:603–11. 10.1016/j.foodchem.2015.07.045 26304389

[2] Farneti B Algarra Alarcón A Papasotiriou FG Samudrala D Cristescu SM Costa G. Chilling-induced changes in aroma volatile profiles in tomato. Food Bioproc Tech. (2015) 8:1442–54. 10.1007/s11947-015-1504-1 26413182 PMC4579789

[3] Zhu D Ren X Wei L Cao X Ge Y Liu H. Collaborative analysis on difference of apple fruits flavour using electronic nose and electronic tongue. Sci Hortic. (2020) 260:108879–87. 10.1016/j.scienta.2019.108879

[4] Barbosa-Pereira L Rojo-Poveda O Ferrocino I Giordano M Zeppa G. Assessment of volatile fingerprint by HS-SPME/GC-qMS and E-nose for the classification of cocoa bean shells using chemometrics. Food Res Int. (2019) 123:684–96. 10.1016/j.foodres.2019.05.041 31285018

[5] Shen H Tao J. Applying electronic nose based on odour classification and identification technology in detecting the shelf life of fresh fruits. Chem Eng Trans. (2018) 68:217–22. 10.3303/CET1868037

[6] Wilson AD Baietto M. Applications and advances in electronic-nose technologies. Sensors. (2009) 9:5099–148. 10.3390/s90705099 22346690 PMC3274163

[7] Wang L Vestrheim S. Controlled atmosphere storage of sweet cherries (*Prunus avium* L.). Acta Agric Scand B Soil Plant Sci. (2002) 52:136–42. 10.1080/09064710310000482529312407

[8] Tian SP Jiang AL Xu Y Wang YS. Responses of physiology and quality of sweet cherry fruit to different atmospheres in storage. Food Chem. (2004) 87:43–9. 10.1016/j.foodchem.2003.10.014

[9] Goliáš J Němcová A Caněk A Kolenčíková D. Storage of sweet cherries in low oxygen and high carbon dioxide atmospheres. Hortic Sci. (2007) 34:26–34. 10.17221/1843-HORTSCI

[10] Chockchaisawasdee S Golding JB Vuong QV Papoutsis K Stathopoulos CE. Sweet cherry: composition, postharvest preservation, processing and trends for its future use. Trends Food Sci Technol. (2016) 55:72–83. 10.1016/j.tifs.2016.07.002

[11] Vavoura MV Badeka AV Kontakos S Kontominas MG. Characterization of four popular sweet cherry cultivars grown in Greece by volatile compound and physicochemical data analysis and sensory evaluation. Molecules. (2015) 20:1922–40. 10.3390/molecules20021922 25629454 PMC6272425

[12] Cozzolino R Martignetti A Cefola M Pace B Capotorto I De Giulio B. Volatile metabolites, quality and sensory parameters of “Ferrovia” sweet cherry cold stored in air or packed in high CO_2_ modified atmospheres. Food Chem. (2019) 286:659–68. 10.1016/j.foodchem.2019.02.022 30827661

[13] Pace B Capotorto I Cefola M Minasi P Montemurro N Carbone V. Evaluation of quality, phenolic and carotenoid composition of fresh-cut purple Polignano carrots stored in modified atmosphere. J Food Compos Anal. (2020) 86:103363–70. 10.1016/j.jfca.2019.103363

[14] Pinto L Palma A Cefola M Pace B D'Aquino S Carboni C. Effect of modified atmosphere packaging (MAP) and gaseous ozone pre-packaging treatment on the physico-chemical, microbiological and sensory quality of small berry fruit. Food Packag Shelf Life. (2020) 26:100573–82. 10.1016/j.fpsl.2020.100573

[15] Martinez-Sanchez A Tudela JA Luna C Allende A Gil MI. Low oxygen levels and light exposure affect quality of fresh-cut Romaine lettuce. Postharvest Biol Tech. (2011) 59:34–42. 10.1016/j.postharvbio.2010.07.005

[16] Cefola M Pace B Buttaro D Santamaria P Serio F. Postharvest evaluation of soilless-grown table grape during storage in modified atmosphere. J Sci Food Agric. (2011) 91:2153–9. 10.1002/jsfa.4432 21538370

[17] Singleton VL Rossi JA. Colorimetry of total phenolics with phosphomolybdic-phosphotungstic acid reagents. Am J Enol Vitic. (1965) 16:144–58.

[18] Bugatti V Cefola M Montemurro N Palumbo M Quintieri L Pace B. Combined effect of active packaging of polyethylene filled with a nano-carrier of salicylate and modified atmosphere to improve the shelf life of fresh blueberries. Nanomaterials. (2020) 10:2513–26. 10.3390/nano10122513 33327664 PMC7765150

[19] Buysse J Merckx R. An improved colorimetric method to quantify sugar content of plant tissue. J Exp Bot. (1993) 44:1627–9. 10.1093/jxb/44.10.1627

[20] Shi J Nian Y Da D Xu X Zhou G Zhao D. Characterization of flavor volatile compounds in sauce spareribs by gas chromatography–mass spectrometry and electronic nose. LWT. (2020) 124:109182–90. 10.1016/j.lwt.2020.109182

[21] Stocchero M Locci E d'Aloja E Nioi M Baraldi E Giordano G. PLS2 in metabolomics. Metabolites. (2019) 9:51. 10.3390/metabo9030051 30884746 PMC6468483

