# Supplementary material for: Electronic-Nose as Non-destructive Tool to Discriminate “Ferrovia” Sweet Cherries Cold Stored in Air or Packed in High CO2 Modified Atmospheres
Source: Front Nutr. 2021 Sep 21;8:720092. doi: 10.3389/fnut.2021.720092 (PMC8491769; doi:10.3389/fnut.2021.720092)
Supplement: Supplementary file 1 [file Table_1.DOCX]

Table S1. Effect of High-CO_2_ (16% O_2_ + 20% CO_2_ + 64% N_2_) and Air storage up to 21 days at 5 °C on VOCs from sweet cherries (*Prunus avium* cv Ferrovia) analyzed by HS SPME GC-MS.

| **Volatiles** | **Code** | **Fresh** | **CTRL14d** | **CTRL21d** | **HCO_2_14d** | **HCO_2_-21d** | **P** |
| --- | --- | --- | --- | --- | --- | --- | --- |
| **Esters** |  |  |  |  |  |  |  |
| Ethyl acetate | E1 | 0.00 b | 0.00 b | 0.00 b | 0.00 b | 5.37 a | **** |
| Ethyl 2-butenoate | E2 | 0.00 b | 0.00 b | 0.00 b | 0.00 b | 2.39 a | * |
| Ethyl hexanoate | E3 | 0.00 b | 0.00 b | 0.00 b | 0.00 b | 4.11 a | ** |
| 1-Hexyl acetate | E4 | 2.42 ns | 1.16 ns | 1.21 ns | 1.83 ns | 1.29 ns | ns |
| 2-Hexen-1-ol acetate | E5 | 7.58 a | 3.34 b | 2.37 b | 4.10 ab | 2.10 b | * |
| 2-Hexenyl butyrate | E6 | 1.51 b | 1.52 b | 0.70 bc | 2.51 a | 0.00 c | ** |
| Ethyl benzoate | E7 | 0.00 b | 0.00 b | 0.00 b | 0.00 b | 1.61 a | ** |
| *trans* 2-Hexenyl hexenoate | E8 | 0.92 ns | 0.90 ns | 0.00 ns | 0.00 ns | 0.00 ns | ns |
| 2-Hexenyl tiglate | E9 | 0.98 ab | 1.28 a | 0.80 ab | 0.00 b | 0.00 b | * |
| Isopropyl laurate | E10 | 2.17 a | 0.94 bc | 1.33 ab | 0.00 c | 0.00 c | * |
| **Alcohols** |  |  |  |  |  |  |  |
| 1-Penten-3-ol | Al1 | 8.14 a | 3.79 b | 3.34 b | 3.35 b | 0.00 c | *** |
| 3-Hexanol | Al2 | 0.00 b | 2.31 a | 0.00 b | 0.00 b | 0.00 b | ** |
| 1-Pentanol | Al3 | 0.00 b | 0.00 b | 0.00 b | 1.94 b | 7.64 a | **** |
| 3-Methyl-3-buten-1-ol | Al4 | 0.00 b | 3.63 a | 4.88 a | 3.60 a | 4.29 a | * |
| *cis* 2-Penten-1-ol | Al5 | 7.14 a | 0.00 b | 0.00 b | 0.00 b | 0.00 b | **** |
| 3-Methyl-2-buten-1-ol | Al6 | 0.00 b | 7.22 a | 9.10 a | 4.11 ab | 5.77 ab | * |
| 1-Hexanol | Al7 | 165.87 ns | 121.84 ns | 103.00 ns | 171.08 ns | 168.61 ns | ns |
| *trans* 3-Hexen-1-ol | Al8 | 6.07 ns | 3.93 ns | 4.26 ns | 5.00 ns | 8.60 ns | ns |
| *cis* 3-Hexen-1-ol | Al9 | 2.27 ns | 1.88 ns | 3.31 ns | 2.01 ns | 3.19 ns | ns |
| cis 2-Hexen-1-ol | Al10 | 718.37 ns | 535.62 ns | 448.55 ns | 560.96 ns | 469.20 ns | ns |
| 1-Octanol | Al11 | 1.64 ns | 2.37 ns | 1.76 ns | 2.40 ns | 2.11 ns | ns |
| Nonanol | Al12 | 2.42 ns | 1.65 b | 0.00 c | 1.01 bc | 0.89 ns | * |
| Benzene methanol | Al13 | 4.59 ns | 5.64 ns | 12.25 ns | 9.17 ns | 11.62 ns | ns |
| 1-Dodecanol | Al14 | 5.38 ns | 6.73 ns | 2.89 ns | 5.87 ns | 2.86 ns | ns |
| **Aldehydes** |  |  |  |  |  |  |  |
| 3-Methyl butanal | Ald1 | 0.00 ns | 0.00 ns | 0.00 ns | 0.20 ns | 0.00 ns | ns |
| Hexanal | Ald2 | 112.31 a | 66.40 ab | 40.52 ab | 42.39 ab | 26.35 b | * |
| 2-Hexenal | Ald3 | 366.64 a | 169.22 b | 125.18 b | 122.45 b | 99.92 b | * |
| Octanal | Ald4 | 0.72 ab | 0.88 a | 0.00 b | 1.11 a | 0.70 ab | ns |
| Nonanal | Ald5 | 6.25 ns | 9.97 ns | 6.21 ns | 8.98 ns | 6.08 ns | ns |
| Decanal | Ald6 | 1.76 ab | 1.93 a | 1.93 a | 0.86 ab | 0.49 b | **** |
| Benzaldehyde | Ald7 | 12.77 ns | 7.16 ns | 10.50 ns | 22.44 ns | 22.74 ns | ns |
| Dodecanal | Ald8 | 6.05 ns | 9.73 ns | 8.76 ns | 8.47 ns | 6.79 ns | ns |
| Tetradecanal | Ald9 | 1.69 ab | 2.43 a | 2.62 a | 2.04 a | 0.00b | * |
| **Ketones** |  |  |  |  |  |  |  |
| 3-Pentanone | K1 | 12.38 a | 0.00 b | 0.00 b | 0.00 b | 0.00 b | *** |
| 2 pentanone 4 methyl | K2 | 3.88 c | 6.49 bc | 8.16 bc | 9.44 ab | 13.04 a | * |
| 1-Penten-3-one | K3 | 2.38 a | 0.00 b | 0.00 b | 0.00 b | 0.00 b | **** |
| γ Butyrolactone | K4 | 0.00 b | 0.00 b | 0.00 b | 0.00 b | 0.82 a | **** |
| 2-Dodecanone | K5 | 0.65 a | 0.65 a | 0.46 ab | 0.00 b | 0.00 b | * |
| **Terpenes** |  |  |  |  |  |  |  |
| dl-Limonene | T1 | 0.74 a | 0.68 a | 0.73 a | 0.00 b | 0.00 b | ** |
| Ocymene | T2 | 1.06 a | 0.00 b | 0.00 b | 0.00 b | 0.00 b | **** |
| Linalool | T3 | 2.75 a | 1.44 ab | 1.46 ab | 0.89 b | 1.83 ab | * |
| α Terpineol | T4 | 1.22 a | 0.88 ab | 0.75 ab | 0.00 b | 1.35 a | * |
| **Others** |  |  |  |  |  |  |  |
| 2-Methylfuran | O1 | 7.60 a | 0.00 b | 0.00 b | 0.00 b | 0.00 b | *** |
| Formammide N,N-dibutyl | O2 | 1.43 a | 0.77 ab | 0.54 b | 0.77 ab | 0.71 b | **** |
| Benzothiazole | O3 | 1.25 ns | 1.33 ns | 1.13 ns | 0.88 ns | 1.27 ns | **** |
